# Relationship between sleep disorders and attention-deficit–hyperactivity disorder in children

**DOI:** 10.3389/fped.2022.919572

**Published:** 2022-07-22

**Authors:** Huimei Yin, Dong Yang, Lin Yang, Guangsheng Wu

**Affiliations:** The Affiliated Hospital of Hangzhou Normal University, Hangzhou, China

**Keywords:** attention-deficit hyperactivity disorder, sleep disorder, Children's Sleep Disturbance Scale, Swanson Nolan and Pelham Version IV Scale, children

## Abstract

**Objective:**

To explore the correlation between sleep disorders and attention-deficit–hyperactivity disorder (ADHD) in children.

**Methods:**

We studied 100 Chinese children (70 boys and 30 girls; mean age, 8.77 ± 2.39 years). Parents completed the Children's Sleep Disturbance Scale (SDSC) and the Swanson Nolan and Pelham Version IV Scale (SNAP-IV) questionnaires. SNAP-IV and SDSC scores were compared in children with and without sleep disorders and ADHD.

**Results:**

There were significant differences in SDSC scores, Arousal Disorder (AD) scores, and Sleep Breathing Disorder (SBD) scores between children with and without ADHD (*P* < 0.05). The sleep disorder group had higher SNAP-IV scores than the non-sleep disorder group (*P* < 0.05). Children with sleep disorders showed higher ADHD symptom values (inattention, hyperactivity/impulsivity, and oppositional defiance) than children without sleep disorders (*P* < 0.01). There was a moderate correlation between SDSC scores and SNAP-IV scores (r = 0.486, *P* < 0.05). Using SNAP-IV scores as the dependent variable, multiple linear regression analysis was applied, and a statistically significant effect of AD and Sleep–Wake Transition Disorder (SWTD) scores on SNAP-IV scores was found (*P* < 0.05). The area under the curve (95% CI) of the SDSC score for predicting sleep disorders with ADHD was 0.714 (0.606, 0.821; *P* = 0.0005).

**Conclusion:**

Children with ADHD are prone to sleep disorders. The higher the ADHD symptom score, the more sleeping problems. Sleep disorders can also cause or exacerbate ADHD symptoms, and the ADHD symptom score correlates with sleep disorder severity. We can reduce the severity of attention-deficit–hyperactivity in children with ADHD by improving their sleep with behavioral sleep interventions.

## Introduction

Sleep is important for bodily repair and memory integration, as well as for brain information processing and emotional regulation. And they all have significant impacts on children's physical development, cognitive development, emotional regulation, attention, behavior, metabolism, and immune function (1). The incidence of sleep disorders in children is ~25%, with 25 to 50% of preschool children and 40% of adolescents having some degree of a sleep problem (2, 3). Attention-deficit–hyperactivity disorder (ADHD), a childhood neurodevelopmental disorder, is one of the most common disorders in this stage of life. It is a highly heterogeneous clinical syndrome caused by a combination of biological, social, and psychological factors and characterized by three nuclear symptoms, namely, inattention, impulsivity, and hyperactivity, which are accompanied by learning difficulties (4). Furthermore, ~30% of children with ADHD have different types or degrees of the disorder (5). Sleep disorders are associated with behavioral disorders (6), attention disorders, irritability, emotional instability, and low tolerance to frustration, all of which contribute to an increased risk of ADHD (7). Although the etiology of ADHD is multifactorial, a growing number of studies suggest that there may be a common underlying neurological etiology between ADHD and sleep disorders, which are mutually causal (8). Therefore, our objective was to investigate the correlation between ADHD and sleep disorders in children, so as to improve the core symptoms and sleep disorders of children with ADHD through early comprehensive intervention, to promote the recovery of cognitive and social function in children, and to improve their quality of life.

## Methods

### Participants

The group consisted of 100 children (age, 4–16 years) who visited the Children's Cognitive Sleep Clinic and Child Health Clinic from September 2020 to December 2021 at the Affiliated Hospital of Hangzhou Normal University. Some children presented with ADHD, and the children were diagnosed for the first time and had never been evaluated for psychiatric disorders or treated with psychopharmacological medicines. Other children visited the hospital for other reasons. The exclusion criteria were as follows: overweight and obese children; children with extensive developmental disorders, severe physical diseases, organic diseases of the nervous system, and other mental illnesses; other diseases that seriously affect sleep quality such as acute attacks of bronchial asthma, allergic rhinitis, urticaria, and respiratory tract infections; and caregivers that failed to complete the questionnaires. This study was approved by the Medical Ethics Committee of the Affiliated Hospital of Hangzhou Normal University [approval number: 2022(E2)-KS-041]. Oral informed consent was obtained from the parents of all participants.

### Assessment of sleep disturbances

Sleep disturbances were measured with the Sleep Disturbance Scale for Children (SDSC) questionnaire (9), a well-validated parental-report instrument. Parents were asked to answer 26 questions that were grouped into six sleep factors, namely, Behavioral Sleep problems of Initiating and Maintaining Sleep (BSP); Sleep Breathing Disorders (SBD); Arousal Disorders (AD); and Sleep–Wake Transition Disorders (SWTD), such as sleepwalking, sleep talking, and bruxism; Excessive Daytime Somnolence (EDS); Sleep Hyperhidrosis—night sweating (HYH); and Total Sleep Problems (TSP), which resulted in the sum of all individual question scores. The SDSC questionnaire is reliable and useful in screening for parent-reported sleep disorders in Chinese children (Cronbach's α = 0.81), and children were considered to have sleep disorders when the total score of the SDSC questionnaire was > 39 (10). The total score and the scores of the six dimensions indicated the degree of the sleep disorder.

### Screening for the diagnosis of ADHD

The Swanson Nolan and Pelham Version IV Scale (SNAP-IV) for parents was used for the diagnosis of ADHD (11). The SNAP-IV questionnaire is frequently applied to clinical trials for the assessment of ADHD treatment (12, 13). Given that the SNAP-IV questionnaire has items for ADHD (18 items) and oppositional defiant disorder (8 items), clinicians can assess not only a patient's hyperactivity/impulsivity and inattention, but also the behavioral symptoms of the most frequently observed co-morbid behavioral disorders in a patient with ADHD. The Chinese version of SNAP-IV questionnaire is a reliable and valid instrument for rating ADHD and Oppositional Defiant Disorder (ODD) symptoms (14, 15). Symptoms are scored on 4-point Likert-scale (from 0 = not at all to 3 = very much). Subscale scores are calculated by averaging the item scores within the domains of Inattention, Hyperactivity/Impulsivity, and Opposition/Defiance. The cutoff value for the SNAP-IV is a subscale mean score > 1, and subscale scores indicated the degree of ADHD.

### Statistical analyses

Continuous variables were expressed as mean ± standard deviation (SD), whereas categorical variables were expressed as percentage. Normality was confirmed for all variables by histograms and Q–Q plots. Differences between cases and controls were analyzed with independent samples *t*-tests or chi-square tests. Associations of SNAP-IV scores with SDSC scores were analyzed using Pearson correlation analysis and linear regression analysis. Analyses were performed using the SPSS 25.0 statistical software package, and a *P* < 0.05 was considered statistically significant.

## Results

### General characteristics of the participants

A total of 100 children aged 4–16 years with a mean age of 8.77 ± 2.39 years were enrolled in the study, including 70 (70.0%) male children and 30 (30.0%) female children (Table 1). There were 66 children with suspected ADHD screened by the SNAP-IV questionnaire, and there was no significant difference in the male-to-female ratio (*P* = 0.080). There was no significant difference in age between the two groups (*P* = 0.299). Sixty-three children with sleep disorders were screened by the SDSC questionnaire, including 46 (73.0%) male children and 17 (27.0%) female children, and the difference was not statistically significant (*P* = 0.390). The prevalence of sleep disorders in children with and without ADHD was significantly different (*P* = 0.018). There were 35 male and 12 female children with ADHD and sleep disorders, and the difference was not statistically significant (*P* = 0.905).

**Table 1:** General characteristics of the participants.

**General characteristics of** **the participants**	**SNAP-IV (–)**	**SNAP-IV (**+**)**	**T or chi-square**	**DOF**	***P*** **-value**
Total population	34 (34.0%)	66 (66.0%)			
Male	20 (58.8%)	50 (75.8%)	3.064	1	0.080
Female	14 (41.2%)	16 (24.2%)			
Age	9.12 ± 2.63	8.59 ± 2.26	1.044	98	0.299
SD	16 (25.4%)	47 (74.6%)	5.616	1	0.018
Male	11 (23.9%)	35 (76.1%)	0.014	1	0.905
Female	5 (29.4%)	12 (70.6%)			
Without SD	18 (48.6%)	19 (51.4%)			

*SNAP-IV, Swanson Nolan and Pelham Version IV Scale; SDSC, Sleep Disturbance Scale for Children; SD, sleep disturbances*.

### Comparison of SDSC scores and SNAP-IV scores in children

Children with ADHD had significantly higher SDSC scores than children without ADHD (*P* = 0.001), and AD scores and SBD scores also differed significantly in children with and without ADHD (Table 2). Children with sleep disorders had significantly higher SNAP-IV scores than children without sleep disorders (*P* = 0.000). Children with sleep disorders showed higher ADHD symptom values (inattention, hyperactivity/impulsivity, and oppositional defiance) than children without sleep disorders (Table 3).

**Table 2:** Comparison of SDSC scores in children with and without ADHD.

	**SNAP-IV (–)** **(*****n*** = **34)**	**SNAP-IV (**+**)** **(*****n*** = **66)**	***T*** **-value**	**DOF**	***P*** **-value**
SDSC (χ ± s)	40.47 ± 8.17	47.20 ± 9.35	−3.552	98	0.001
BSP (χ ± s)	12.82 ± 3.33	14.20 ± 3.66	−1.831	98	0.070
AD (χ ± s)	3.71 ± 1.61	4.39 ± 1.57	−2.062	98	0.042
EDS (χ ± s)	6.00 ± 2.62	6.82 ± 3.86	−1.252	90.610	0.214
SBD (χ ± s)	5.50 ± 2.77	7.45 ± 3.73	−2.961	85.633	0.004
SWTD (χ ± s)	8.03 ± 2.59	9.26 ± 3.17	−1.949	98	0.054
HYH (χ ± s)	4.32 ± 2.04	5.08 ± 2.15	−1.685	98	0.095

*BSP, Behavioral Sleep problems of Initiating and Maintaining Sleep; AD, Arousal Disorders; EDS, Excessive Daytime Somnolence; SBD, Sleep Breathing Disorders; SWTD, Sleep–Wake Transition Disorders; HYH, Sleep Hyperhidrosis*.

**Table 3:** Comparison of SNAP-IV scores in children with and without SD.

	**Without SD** **(*****n*** = **37)**	**With SD** **(*****n*** = **63)**	***T*** **-value**	**DOF**	***P*** **-value**
SNAP-IV scores (χ ± s)	22.70 ± 10.11	32.46 ± 14.29	−3.983	94.526	0.000
Inattention	9.51 ± 4.74	13.29 ± 5.76	−3.370	98	0.001
Hyperactivity/impulsivity	6.57 ± 4.62	10.05 ± 6.61	−3.088	94.899	0.003
Oppositional defiance	6.32 ± 3.71	9.13 ± 4.73	−3.288	90.052	0.001

### SDSC scores are associated with ADHD symptomatology in children

We identified significant correlations between SDSC scores and ADHD hyperactivity symptoms (r = 0.486, *p* = 0.000). We noted that all SDSC subscales, except SBD, were significantly correlated with ADHD symptoms according to both SNAP-IV and SDSC scales (Table 4). Using SNAP-IV scores as the dependent variable and BSP, AD, EDS, SWTD, and HYH as the independent variables, multiple linear regression analysis was applied and AD and SWTD significantly affected SNAP-IV scores (Table 5).

**Table 4:** Pearson correlation analysis between SNAP-IV scores and SDSC scores.

	**Correlation** **coefficient r**	***P*** **-value**
SDSC scores	0.486	0.000
BSP	0.265	0.008
AD	0.287	0.004
EDS	0.342	0.001
SBD	0.048	0.638
SWTD	0.433	0.000
HYH	0.258	0.009

**Table 5:** Multiple linear regression model for SNAP-IV scores.

**Variable**	**B**	**Standard** **error**	**Standard** **coefficient**	**T value**	***P*** **-value**
BSP	−0.019	0.408	−0.005	−0.045	0.964
AD	1.921	0.781	0.225	2.459	0.016
EDS	0.601	0.445	0.153	1.352	0.180
SWTD	1.258	0.508	0.278	2.477	0.015
HYH	0.577	0.634	0.090	0.910	0.365

*R^2^ = 0.220, Durbin-Watson = 1.685, F = 6.587, P = 0.000, VIF < 5, the independent variables were not collinear and the residuals obeyed a normal distribution*.

### Receiver operating characteristic curve of the SDSC score

**Figure 1:**
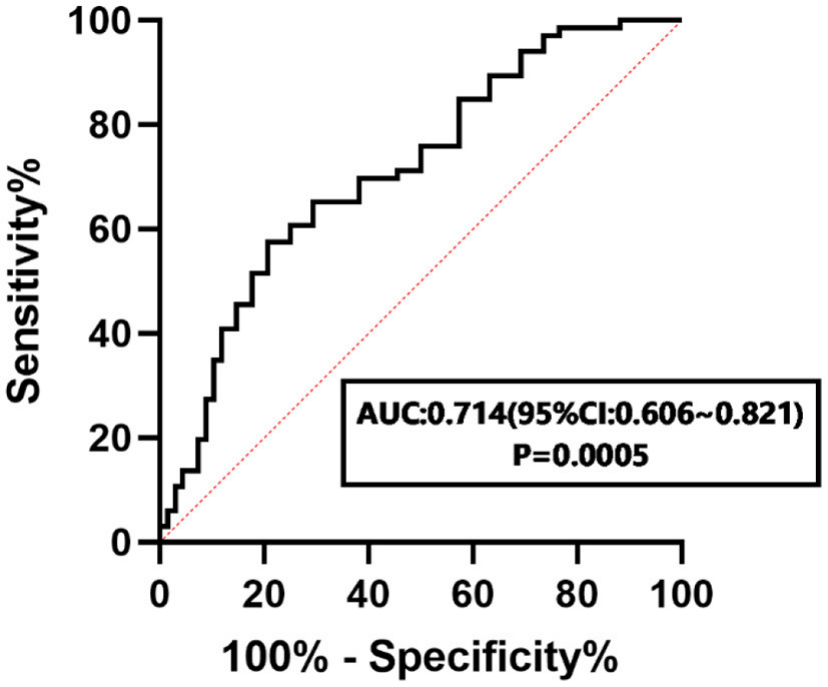
ROC curve of the SDSC score in predicting sleep disorders with ADHD.

The area under the curve (95% CI) of the SDSC score for predicting sleep disorders with ADHD was 0.714 (0.606, 0.821), as shown in Figure 1. The specificity and sensitivity were 0.794 and 0.576, respectively.

## Discussion

Sleep disorders are a common health problem in children, and ADHD is one of the non-negligible diseases among many co-morbid sleep disorders in children. In this study, we investigated the association between ADHD and sleep disorders in children by comparing SDSC scores between children with and without ADHD. The global prevalence of ADHD ranges from 1.2 to 7.3%, with the disorder being 3 to 9 times more common in males than females (16). The prevalence of sleep disturbance symptoms also varies according to gender, and the prevalence of parent-reported sleep disorder symptoms was higher in boys aged 6–14 years (5.19%) than in girls of the same age (3.67%) (10). Although our results showed that ADHD was not significantly higher in male than in female children, there was no significant difference between male and female children with sleep disorders, and further studies are needed to verify the effect of gender on ADHD, as well as sleep disorders, in the future. The incidence of sleep disorders in children with and without ADHD was significantly different. The prevalence of sleep disorders in children with ADHD ranges from 35 to 70% and varies because of gender, age, ADHD subtype, psychiatric co-morbidities, and medication use (17). In this study, the incidence of children with ADHD and co-morbid sleep disorders was 74.6%, confirming the prevalence of sleep disorders in children with ADHD. However, the incidence of sleep disorders was 25.4% in children without ADHD, which was much higher than the previously reported incidence of 4.43% in China (10), and similar to the previously reported incidence of 26.7% in other countries (18). This may be related to the increased attention of parents to children's sleep problems in recent years in China.

The SDSC scores of children with ADHD in our study were significantly higher than those of children without ADHD, and AD and SBD scores were statistically different, confirming that children with ADHD had more sleep problems. Correlation analysis between SNAP-IV and SDSC scores suggests that the two increased linearly, indicating that the higher the ADHD symptom scores, the more sleep problems. Behavioral disruptions related to ADHD may affect sleep at night with symptoms of insomnia, struggling before falling sleep, poor sleep quality, or insufficient sleep duration (19). Zerón et al. reported that ADHD inattentive patients show significant behavioral sleep problems in initiating and maintaining sleep, sleep–wake transition disorders, and excessive daytime somnolence, while ADHD combined-type patients show more significant sleep breathing disorders and sleep hyperhidrosis (20). Although only AD and SBD scores were significantly higher in children with ADHD than in those without ADHD, this inconsistency may be related to cultural differences between countries or ADHD subtypes. More in-depth studies are needed on the relationship between different subtypes of ADHD and sleep disorders in the future.

ADHD patients with co-morbid sleep problems were found to have more pronounced symptoms of attention deficit and hyperactive-impulsive behavior, as well as oppositional defiance (21–23). Our study also found that children with sleep disorders had higher ADHD symptom scores. There were significant differences in attention deficit, hyperactivity-impulsivity, and oppositional defiance scores compared with children without sleep disorders, suggesting that sleep disturbances can produce and/or exacerbate symptoms of inattention and hyperactivity, which may then produce or exacerbate some sleep problems (24). As such, the two interact, cause-and-effect each other, and share diseases with each other.

Correlation analysis revealed a moderate correlation between SDSC and SNAP-IV scores (r = 0.486), which increased linearly, and ADHD symptom scores increased significantly with increasing SDSC scores, suggesting that ADHD symptoms were associated with sleep disorder severity. This means that we may reduce the severity of inattention and hyperactivity-impulsivity in children with ADHD by improving sleep with behavioral sleep interventions (25, 26). Of course, more in-depth clinical studies are needed in the future.

BSP, AD, EDS, SWTD, and HYH in the SDSC subscales were all significantly associated with ADHD symptoms. In constructing the linear regression model, the effects of AD and SWTD on SNAP-IV scores were statistically significant. This suggests that ADHD and sleep disorders may have similar symptoms and effects on daily functions and quality of life (27). Primary sleep disorders in children may cause daytime neurobehavioral problems similar to ADHD, and sleep disorders may also aggravate ADHD-like symptoms. Thus, in children with sleep disorders, ADHD-like symptoms resemble ADHD (28). Therefore, during the diagnosis and treatment of ADHD, we should attach great importance to whether the child has sleep disorders, while evaluating, intervening, and treating the child to avoid improper psychotropic drug intervention and further complicating the condition (29).

In this study, we used the SNAP-IV questionnaire for ADHD and found that SDSC scores predict sleep disorders with ADHD, and the AUC of the ROC curve was 0.714. This suggests the potential use of SDSC scores as a clinical tool to assist in the diagnosis of ADHD, and future studies with larger sample sizes are needed to verify these findings, which may help in the early intervention and treatment of ADHD associated with sleep disorders.

In conclusion, children with ADHD are prone to sleep disorders, and the higher the ADHD symptom scores, the more sleep problems. Sleep disorders can also cause or exacerbate ADHD symptoms, and ADHD symptom scores correlate with sleep disorder severity. We can reduce the severity of inattention and hyperactivity in children with ADHD by improving sleep with behavioral sleep interventions. A limitation of this study may be found in the lack of an objective measure, which may have provided better information on sleep disorders and allowed a more accurate diagnosis (30). However, actigraphy and polysomnography in children may be poorly tolerated, may increase bed anxiety, and are not always feasible in everyday clinical practice; on the other hand, parental questionnaires like the SDSC have the advantage of saving time and costs, can be administered at each outpatient assessment, and can measure a wide range of sleep parameters in various contexts (31). However, our study design approach had some limitations and is not yet sufficient for the assessment of the causal relationship between ADHD and sleep disturbances. In addition, other sleep parameters, such as sleep duration, sleep onset time, arousal index, apnea hypopnea index (AHI), and circadian parameters, should be further evaluated. Further research is needed on the relationship between ADHD and sleep disorders in children, and the importance of intervening sleep disorders in children to improve ADHD symptoms, which may help to treat the disorder appropriately and effectively.

## Data availability statement

The raw data supporting the conclusions of this article will be made available by the authors, without undue reservation.

## Ethics statement

The studies involving human participants were reviewed and approved by Medical Ethics Committee of the Affiliated Hospital of Hangzhou Normal University. Written informed consent to participate in this study was provided by the participants' legal guardian/next of kin.

## Author contributions

GW and HY designed the study. HY and DY performed research and wrote the paper. HY and LY analyzed data. All authors contributed to the article and approved the submitted version.

## Funding

This study was supported by the Hangzhou Medical Science and Technology Project (A20220128).

## Conflict of interest

The authors declare that the research was conducted in the absence of any commercial or financial relationships that could be construed as a potential conflict of interest.

## Publisher's note

All claims expressed in this article are solely those of the authors and do not necessarily represent those of their affiliated organizations, or those of the publisher, the editors and the reviewers. Any product that may be evaluated in this article, or claim that may be made by its manufacturer, is not guaranteed or endorsed by the publisher.
